# In-house ELISA protocols for capsid p24 detection of diverse HIV isolates

**DOI:** 10.1186/s12985-023-02242-5

**Published:** 2023-11-17

**Authors:** Mariano A. Molina, Monique Vink, Ben Berkhout, Elena Herrera-Carrillo

**Affiliations:** Department of Medical Microbiology, Laboratory of Experimental Virology, Amsterdam UMC, AMC Location, University of Amsterdam, Amsterdam, The Netherlands

**Keywords:** HIV, CA-p24, ELISA, Antibodies, Antigen

## Abstract

**Background:**

The capsid p24 (CA-p24) antigen is a component of the viral capsid of human immunodeficiency virus (HIV) that has been commonly used for clinical diagnosis and monitoring of HIV infections in Enzyme-linked Immunosorbent Assays (ELISAs). Commercial CA-p24 ELISAs are widely used in research settings, but these kits are costly and have limited breadth for detecting diverse HIV isolates.

**Methods:**

Commercial CA-p24 antibodies were used as capture and detection antibodies. Specific CA-p24 ELISAs were established with these antibodies and tested for the detection of HIV-1 isolates with the aim of developing in-house protocols to recognize HIV-1 infections in vitro for research purposes.

**Results:**

Here we present four protocols for in-house ELISAs to detect HIV CA-p24 using commercial antibodies. The assays were able to detect the CA-p24 antigen of different HIV-1 isolates tested. Comparison between the protocols showed that these in-house ELISAs exhibit high specificity, sensitivity, and reproducibility for CA-p24 quantitation but their reactivity varied per HIV-1 isolate and subtype.

**Conclusions:**

These optimized ELISA protocols represent valuable tools to investigate HIV-1 infections in research facilities at a lower price than commercial CA-p24 kits.

**Supplementary Information:**

The online version contains supplementary material available at 10.1186/s12985-023-02242-5.

## Background

HIV infections are prevalent worldwide. If left untreated, HIV can progress to AIDS (Acquired Immunodeficiency Syndrome) and cause more than 600,000 deaths every year [1]. The current standard treatment is combination antiretroviral therapy (cART), which is able to effectively control viral replication [2]. However, cART must be taken by patients for their complete life, it has side-effects, and it is not curative. There is no cure for the disease or effective vaccines against the virus [3, 4]. Hence, there is still a need to understand HIV infection biology and to develop novel therapies against viral infection and reservoir cells (latently infected cells).

Current research approaches include viral genome editing through CRISPR-Cas systems, stem cell transplants, and mRNA vaccines [5–7]. Successful HIV research is greatly dependent on the accurate quantitation of viral infection. HIV capsid CA-p24 antigen testing has been widely used in HIV research [8–11]. The detection of CA-p24 antigen has proven to be reliable, cost-effective, and a quick way to identify HIV infection [12–14]. Commercial CA-p24 ELISA kits can detect CA-p24 in very low concentrations but are generally costly. In addition, mutations in the HIV genome of distinct virus isolates may affect the antibodies binding properties to the CA-p24 antigen and reduce the sensitivity of the assay [15–18]. Thus, robust in-house ELISAs are needed to support research studies on HIV infection biology and potential HIV therapies.

In this study, we aimed to optimize and compare four in-house CA-p24 ELISA systems to detect and quantitate distinct HIV-1 isolates. We determined the reproducibility and sensitivity of the ELISA systems and described their characteristics for testing CA-p24 antigen.

## Results

We have developed four in-house CA-p24 ELISAs using commercial antibodies to identify diverse HIV-1 isolates and detect HIV infections in vitro. These protocols were named based on the commercial source of the antibodies: Aalto Bio Reagents (ABR), Anogen (ANG), Sino Biological (SB), and R&D Systems (RND). The costs per each in-house assay range between €25 and €30 per one 96-well plate, which is 15 × lower than medium-cost commercial kits, 25 × lower than high-cost commercial kits (*q* = 0.0065, Kruskal–Wallis’ test), and 45 × lower than very high-cost commercial kits (*q* = 0.0048) (Additional file 1: Fig. S1 and Additional file 2: Table S1).

To optimize the CA-p24 ELISA protocols, we initially conducted an assessment of our in-house reagents, including PBS buffers and LumiPhos substrates (see Table 1). We also evaluated the performance of our equipment, specifically the BioTek ELx405 Select washer and the GloMax® plate reader. During this process, we determined the ideal working volume for each assay, with most assays showing optimal results using either 25 µL or 50 µL (as indicated in Table 1). Note we have utilized a standardized CA-p24 antigen across all assays to ensure consistency (Tables 3, 4, 5, 6). The working concentrations of antibodies, incubation periods, washing buffers, and blocking buffers were all employed according to the recommendations provided by the manufacturers (see "Methods" and Table 1). Lastly, we assessed the linear range of each assay, resulting in three assays with an upper limit of 5 ng/mL (ABR, ANG, and SB) and one assay with a limit of 3 ng/mL (RND) (Table 1).

**Table 1:** Variations between ELISA protocols

Features	ABR	ANG	SB	RND
Working volume	25 µL	25 µL	25 µL	50 µL
Working concentration of capture antibody	10 µg/mL	10 µg/mL	2 µg/mL	4 µg/mL
Washing buffer post-coating	TBS-based buffer	PBS-based buffer	PBS-based buffer	PBS-based buffer
Blocking step post-coating	No	Yes	Yes	Yes
Working concentration of detection antibody	60 ng/mL	60 ng/mL	100 ng/mL	125 ng/mL
Detection antibody	AP conjugated	AP conjugated	HRP conjugated	Unconjugated
Incubation period post-antibody addition	1 h	1 h	1 h	2 h
Washing buffer post-antibody addition	PBS-based buffer	PBS-based buffer	PBS-based buffer	PBS-based buffer
Additional conjugation	NA	NA	NA	HRP
Substrate reagent	LumiPhos Plus	LumiPhos Plus	LumiPhos-HRP	LumiPhos-HRP
Incubation period post-substrate addition	20 min	20 min	2 min	2 min
Lower range	0.014 ng/mL	0.17 ng/mL	0.018 ng/mL	0.015 ng/mL
Upper range	5 ng/mL	5 ng/mL	5 ng/mL	3 ng/mL

**Fig. 1:**
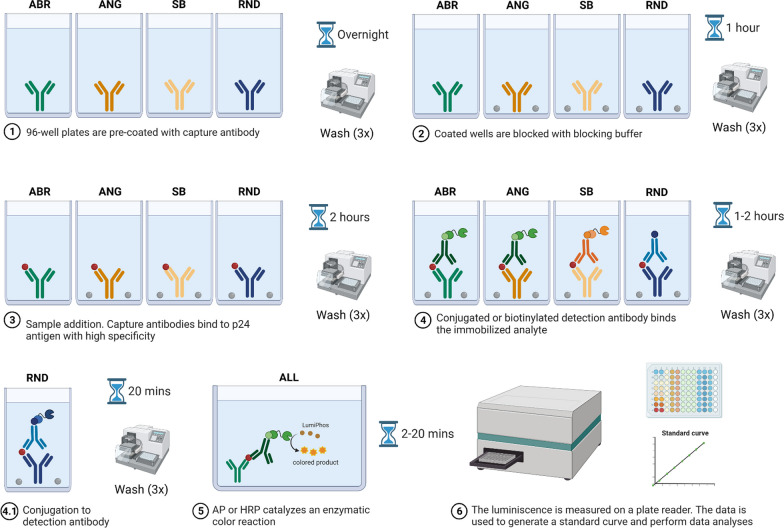
Schematic summary of CA-p24 ELISA protocols. Diagram of in-house CA-p24 ELISA protocols. Complete details of the protocols are described in the Results and Methods sections. ABR = Aalto Bio Reagents; ANG = Anogen; SB = Sino Biological; RND = R&D Systems

Here we described the ELISA protocols optimized in-house (Fig. 1, Table 1) and the in vitro validations performed to determine the sensitivity, specificity, reactivity, and reproducibility of these assays towards distinct HIV-1 isolates.

### CA-p24 ELISA protocols

Half-area white 96-well plates were pre-coated with capture antibody and incubated at room temperature overnight (Fig. 1, Table 1, see “Methods”). Next, plates were washed three times with wash buffer and blocked with blocking buffer. The ABR-protocol does not have an initial blocking step in our protocol and requires a TBS-based washing buffer for this step. After 1 h incubation at room temperature while shaking, the plates were washed. Diluted samples, CA-p24 standard proteins and controls (PBS) were then added to the wells, and the plates were incubated for 2 h at room temperature while shaking. Thereafter, plates were washed, and the alkaline phosphatase (AP)- or horseradish peroxidase (HRP)-conjugated detection antibody (ABR-, ANG-, and SB-protocols) or an unconjugated detection antibody (RND-protocol) was added to the wells. ABR-detection antibody solution with skim milk was used as a blocking step. Following 1–2 h incubation at room temperature while shaking, the plates were washed (Table 1). Note, the ABR protocol requires a PBS-based wash buffer for this step while the RND-protocol needs conjugation with Streptavidin-HRP, followed by incubation at room temperature for 20 min while shaking. Each conjugate (AP or HRP) catalyzed an enzymatic reaction by the addition of LumiPhos Plus or LumiPhos-HRP to the wells. After brief incubation (Table 1) with the LumiPhos solution, the luminescence generated was immediately read on GloMax® plate reader (Fig. 1, see “Methods”). Standard curves were created, and data analyses were performed. One set of standard curves obtained through all tested ELISAs are shown in Additional file 1: Fig. S2. HIV isolates (Table 2) were measured in duplicate by two independent ELISA runs per ELISA protocol.

**Table 2:** List of HIV isolates

HIV isolate	Accession number	Subtype	Tropism
DJ258	L22939	A	R5
92UG029	Partial	A	X4
NSI18	NA	B	R5
SF162	M65024	B	R5
ADA	AF004394	B	R5
Ba-L	M68893	B	R5
JR-CSF	M38429	B	R5
HXB2	MZ868395	B	X4
NL4-3	AF003887	B	X4
SI19	NA	B	X4
SI22	NA	B	X4
LAI	X01762	B	X4
IIIB	KJ925006	B	X4
RF	M17451	B	R5X4
SF2	K02007	B	R5X4
3920C6	Partial	B	R5X4
39201E8	Partial	B	R5X4
PHD79C12	Partial	C	R5
PHD79B8	Partial	C	X4
92UG024	Partial	D	X4
94TH001	Partial	CRF01_AE	R5
94TH304	NA	CRF01_AE	X4

### Detection of HIV-1 isolates

**Fig. 2:**
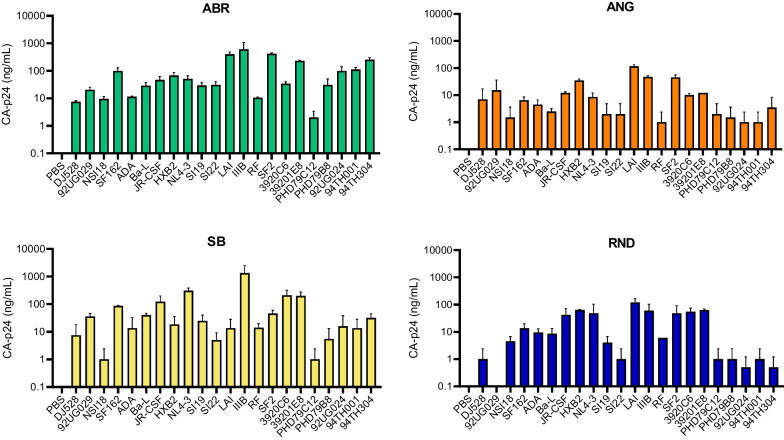
Analyses of CA-p24 antigen concentration on diverse HIV isolates. Twenty-two HIV-1 isolates were tested through ABR-, ANG-, SB-, and RND-CA-p24 ELISAs for detection of CA-p24 antigen. All ELISAs detected most of the isolates, except RND-CA-p24 ELISA which did not detect the 92UG029 isolate. PBS was used as negative control. Values were measured in two independent ELISA runs and error bars represent the standard deviation (SD). The CA-p24 axis is log-scaled in the graph. The list of HIV isolates can be found in Table 1. ABR = Aalto Bio Reagents; ANG = Anogen; SB = Sino Biological; RND = R&D Systems

We have previously used the ABR-CA-p24 ELISA to measure the CA-p24 concentration in HIV infection experiments for over 20 years and therefore considered this assay the standard reference [19–22]. When testing the 22 HIV isolates (Table 2), we observed that the ABR-, ANG-, and SB-CA-p24 ELISAs detected all isolates and only RND-CA-p24 ELISA was unable to detect the isolate 92UG029 (Fig. 2). Overall, higher CA-p24 values were detected by ABR-CA-p24 ELISA in comparison to the other CA-p24 ELISAs. When analyzing the CA-p24 concentrations by the HIV subtype (Table 2), ANG- and RND-CA-p24 ELISAs exhibited low reactivity for detecting non-B subtype isolates (Fig. 2).

### Reactivity of ELISA systems towards HIV-1 A and B subtypes

**Fig. 3:**
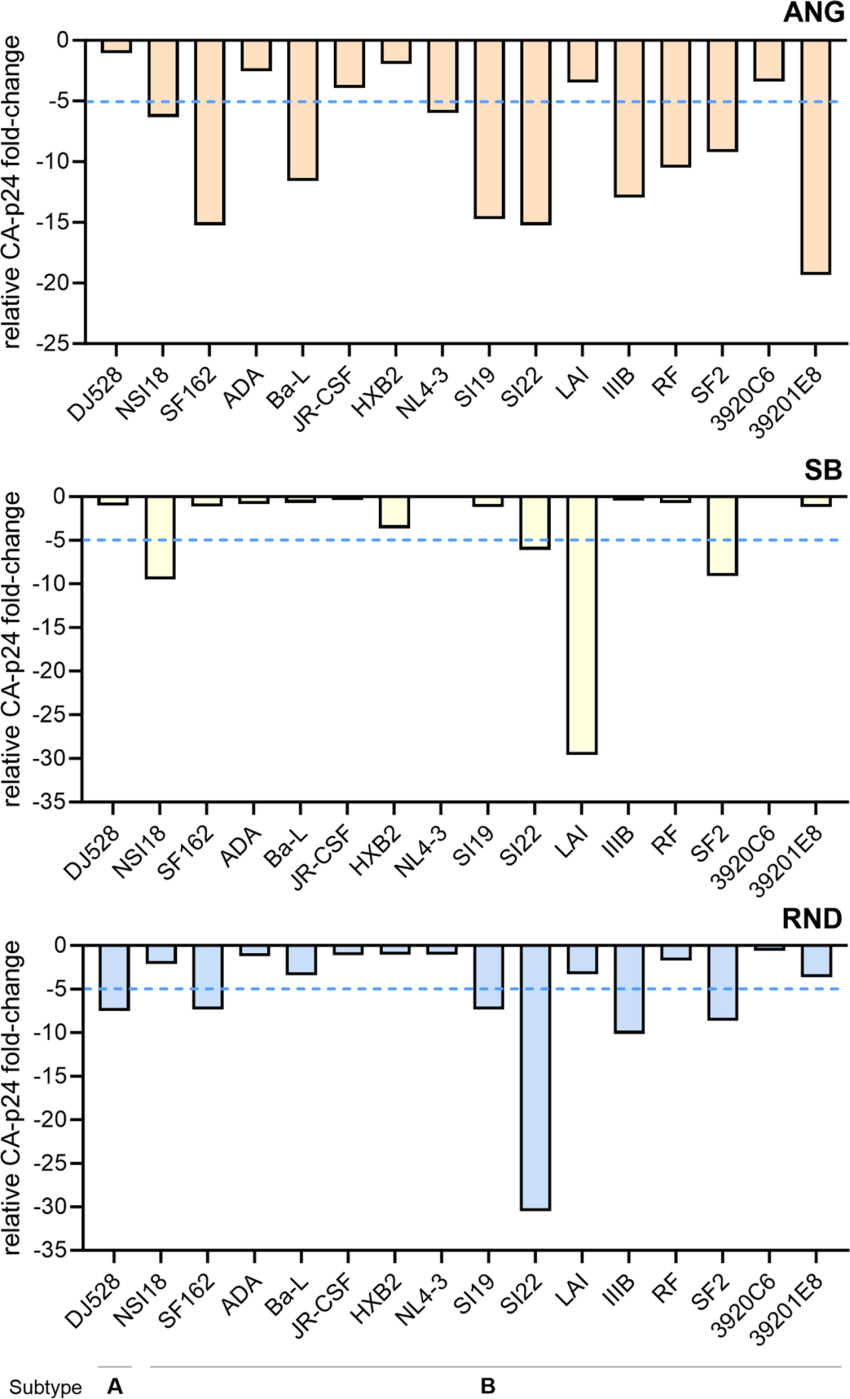
Fold-change in CA-p24 detection of HIV-1 A/B isolates relative to ABR-CA-p24 ELISA. ANG-CA-p24 ELISA exhibits low reactivity for HIV-1 A and B subtypes when compared to ABR-CA-p24 ELISA. RND-CA-p24 ELISA shows acceptable reactivity for HIV-1 A and B subtypes when compared to ABR-CA-p24 ELISA, but low reactivity for the SI22 isolate. SB-CA-p24 ELISA displays good reactivity for HIV-1 A and B subtypes when compared to ABR-CA-p24 ELISA, but low reactivity for the LAI isolate. ABR = Aalto Bio Reagents; ANG = Anogen; SB = Sino Biological; RND = R&D Systems

We also observed that the measured CA-p24 concentrations of several HIV-1 isolates differed with each ELISA system (Fig. 2) and therefore calculated the CA-p24 fold-change of ANG-, SB-, and RND-CA-p24 ELISAs relative to the concentrations obtained by ABR-CA-p24 ELISA to determine their reactivity towards each isolate (Fig. 3). We focused the analyses on HIV-1 A and B subtypes because all systems performed well on these isolates (Fig. 2). Compared to ABR-CA-p24 ELISA, we observed that ANG-CA-p24 ELISA showed the lowest reactivity towards the isolates, detecting 10 out of 16 isolates (62.5%) with more than a fivefold reduction in CA-p24 concentration (Fig. 3). Overall, RND-CA-p24 ELISA exhibited a better reactivity towards most isolates when compared to ANG-CA-p24 ELISA, however, the system still showed poor reactivity when compared to ABR-CA-p24 ELISA by detecting 6 out of 16 isolates (37.5%) with more than a fivefold reduction in CA-p24 concentration (Fig. 3). RND-CA-p24 ELISA also detected the isolate SI22 with more than a 25-fold reduction in CA-p24 concentration when compared to ABR-CA-p24 ELISA, suggesting a very low reactivity of this system towards this isolate. SB-CA-p24 ELISA showed better reactivity towards the isolates when compared to ANG- and RND-CA-p24 ELISA by detecting only 4 out of 16 isolates (25%) with more than a fivefold reduction in CA-p24 concentration (Fig. 3). However, SB-CA-p24 ELISA detected the LAI isolate with more than a 25-fold reduction in CA-p24 concentration when compared to ABR-CA-p24 ELISA, implying very low reactivity of this assay for this particular isolate.

### Correlations between ELISA systems

**Fig. 4:**
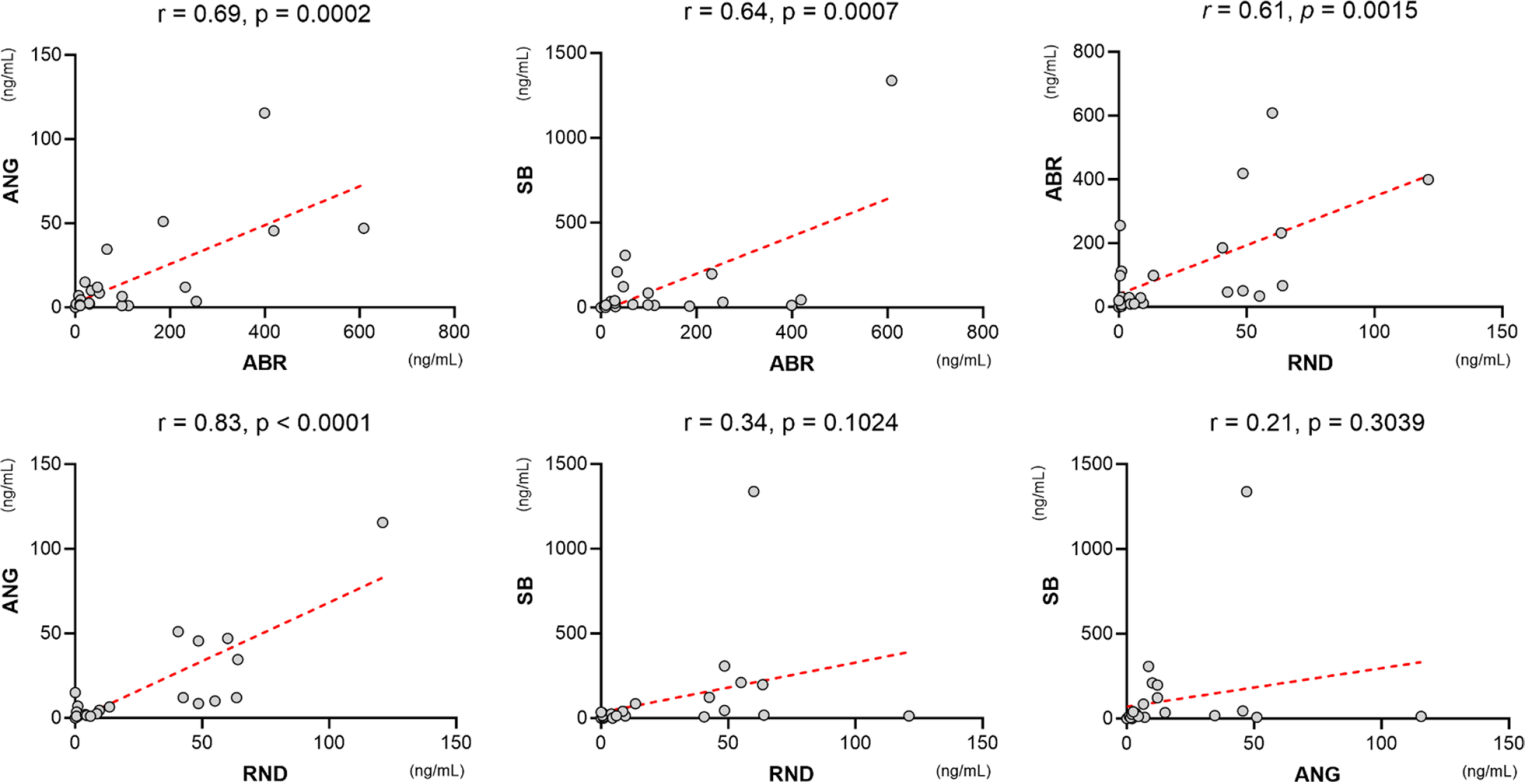
Comparative reactivity towards HIV-1 isolates between assays as determined by Pearson’s *r* correlation. ANG-, SB-, and RND-CA-p24 ELISAs exhibit a significantly high correlation with ABR-CA-p24 ELISA. ANG-CA-p24 ELISA exhibits a significantly high correlation with RND-CA-p24 ELISA, whereas both RND- and ANG-CA-p24 ELISA show a low correlation with SB-CA-p24 ELISA. Concentrations of CA-p24 are displayed in ng/mL. ABR = Aalto Bio Reagents; ANG = Anogen; SB = Sino Biological; RND = R&D Systems

To further test the reactivity of the ANG-, SB-, and RND-CA-p24 ELISAs when compared to our standard ABR-CA-p24 ELISA, we determined the Pearson *r* correlation of the CA-p24 concentrations of all tested isolates measured by each assay (Fig. 4). Here we observed that ANG-CA-p24 ELISA measurements exhibited a significantly high correlation with ABR-CA-p24 ELISA (*r* = 0.69 [95% CI 0.40–0.85], *p* = 0.002), possibly due to the use of the same detection antibody. Similarly, SB-CA-p24 ELISA showed a significantly high correlation with ABR-CA-p24 ELISA (*r* = 0.64 [95% CI 0.31–0.82], *p* = 0.0007). RND-CA-p24 ELISA also exhibited a significant high correlation with ABR-CA-p24 ELISA (*r* = 0.61 [95% CI 0.27–0.81], *p* = 0.0015) (Fig. 4). We found the highest correlation between RND- and ANG-CA-p24 ELISAs (*r* = 0.83 [95% CI, 0.65–0.92], *p* < 0.0001), suggesting a similar sensitivity between both assays, possibly due to their similar reactivity for HIV-1 B subtypes. The lowest correlations were observed between RND- and SB-CA-p24 ELISAs (*r* = 0.34 [95% CI − 0.07 to 0.65], *p* = 0.1024) and between ANG- and SB-CA-p24 ELISAs (*r* = 0.21 [95% CI − 0.20 to 0.57], *p* = 0.3039) (Fig. 4).

### Reproducibility of ELISA systems and influence of viral inactivation

We then assessed the reproducibility of the ELISA systems, examining both their consistency within individual assays and their reliability between different assays. In order to assess within-assay reproducibility, we employed the NL4-3 HIV-1 isolate as a test model. We quantified its concentration in each assay by utilizing technical replicates and applying different inactivation treatments, including heat and/or detergent. We observed that the concentration of the NL4-3 isolate remained consistent between the replicates within the tested conditions (Additional file 1: Fig. S3), and across previous ELISA runs (Fig. 2) indicating that all ELISA systems demonstrate within-assay reproducibility. We observed that most ELISA systems are not affected by the inactivation method, whereas SB and RND protocols showed a significant drop in reactivity when samples were heat-inactivated. However, this effect was not observed when heat inactivation was combined with detergent-based inactivation (Additional file 1: Fig. S3). Additionally, we noted that the SB-CA-p24 ELISA exhibited higher reactivity for NL4-3 HIV-1 compared to other systems, resulting in a higher measured CA-p24 concentration, which is consistent with previous findings (Figs. 2, 3). Since we observed that the reactivity of the assays was mostly isolate-dependent (Figs. 2, 3), we evaluated the between-assay reproducibility using the standard CA-p24 protein (Additional file 1: Fig. S4). By analyzing the concentrations of CA-p24 based on the generated luminescence by assay, we noticed an excellent correlation of all concentrations between assays (Pearson *r* > 0.97, *p* < 0.002), suggesting a high between-assay reproducibility for the ELISA systems.

## Discussion

Overall, all newly developed assays did not outperform the ABR-CA-p24 ELISA in the detection of different HIV-1 isolates. ANG-CA-p24 ELISA exhibited poor reactivity towards the isolates when compared to ABR-CA-p24 ELISA but similar reactivity as RND-CA-p24 ELISA. ANG- and ABR-CA-p24 ELISA protocols only differ in the capture antibody used for coating the plates, which suggests that a low binding affinity of the ANG-capture antibody for particular HIV-1 isolates may be the cause of the low performance of this assay for measuring CA-p24 antigen.

SB-CA-p24 ELISA showed better reactivity towards the HIV-1 isolates when compared to RND- and ANG-CA-p24 ELISAs. However, SB-CA-p24 ELISA exhibits low reactivity for the HIV-1 LAI isolate, which is one of the most commonly used isolates for in vitro experiments [19, 23–25], but a high reactivity for the lab-adapted HIV-1 NL4-3 strain. Interestingly, the capsid protein of HIV-1 NL4-3 and LAI isolates only differ in four amino acids: L6I, L83V, H120N and G208A, and the residues 6, 83, and 120 are among the most polymorphic positions within the capsid protein [26–28]. It is possible that these substitutions may cause a reduced affinity of the SB antibodies for the LAI CA-p24 antigen. However, additional studies are needed to identify whether these mutations may cause lower binding affinities of the tested antibodies and they should be considered when developing CA-p24 immunoassays. The CA-p24 concentrations measured with the RND-CA-p24 ELISA correlated with the values obtained with ABR- and ANG-CA-p24 ELISAs, demonstrating the suitability of this assay for in vitro research as previously reported [29].

The strengths of this study and the CA-p24 ELISA protocols are the development of low-cost in-house assays to detect HIV-1 in vitro and with high sensitivity for diverse HIV-1 isolates and subtypes. Potential limitations may include a relatively low number of tested HIV-1 isolates [30]. Of note, we used the same CA-p24 antigen to generate the standard curves and properly perform comparison analyses between the ELISA systems. The obtained linear regression curves (Additional file 1: Fig. S2) showed fitted lines with high correlation between the OD values and CA-p24 concentrations and small residuals, demonstrating that the assays detected accurately the CA-p24 antigen. Further tests may also involve comparison analysis with RT-qPCR [31], and other immunoassays [32, 33].

## Conclusions

The development of in-house ELISA protocols for detecting the CA-p24 of diverse HIV-1 isolates represents a valuable contribution to the field of HIV research. The protocols presented in this study provide a cost-effective alternative for detecting CA-p24 and have been shown to exhibit high sensitivity and specificity. Moreover, identifying CA-p24 in diverse HIV-1 isolates allows for detecting a broad range of viral subtypes, which is essential for accurately recognizing and monitoring HIV infections in vitro. The availability of these protocols for in-house development represents a significant tool for researchers worldwide.

## Methods

### Biosafety

The National Institute for Public Health and the Environment (RIVM) guidelines classify HIV-1 as a Risk Group 3 agent. Research involving HIV-1 was approved by the institutional biosafety office and carried out according to laboratory biosafety 3 guidelines.

### Production of HIV-1 isolates

Twenty-two different HIV-1 isolates were produced according to standard protocols [34]. The HIV-1 panel of 22 strains includes strains belonging to subtypes A, B, C, D and circulating recombinant form (CRF) 01_AE (Table 2). We also tested CXCR4 (X4), CCR5 (R5) or dual-tropic (R5X4) strains. The viruses listed in Table 2 were obtained from the NIH AIDS Research and Reference Program (Division of AIDS, NIAID, NIH) or from HIV-infected patients in Ethiopia and the Netherlands. Two biological clones derived from ACH0039 were included in this study (3920C6 and 39201E8) [35]. PHD79B8 and PHD79C12 viruses were isolated from the Ethiopian individual PHD79 [36]. HIV-1 molecular clones were transfected in human embryonic kidney (HEK) 293 T cells for virus production as described previously [24]. Cell-free viruses were passed through 0.2 μm pore-size filters and stored in aliquots at − 80 °C.

Virus stocks were generated on CD8 + -depleted human peripheral blood mononuclear cells (PBMCs) for each isolate, including the lab-adapted strains, and virus production was measured by CA-p24 ELISA (in-house assays). The HIV stocks were inactivated with 0.1% Empigen (Sigma-Aldrich, Cat. No. 45165) followed by incubation at 56 °C for 30 min.

### CA-p24 ELISA solutions

1. Aalto Bio Reagents (ABR) system

- First wash buffer solution and dilution buffer:
  - 10 × Tris buffered saline (TBS) buffer: 1.37 M NaCl (80 g) + 27 mM KCl (2 g) + 2.4 g of Tris in a volume of 950 ml of ddH_2_O. Adjust pH to 7.4 with HCl and add ddH_2_O up to a volume of 1,000 mL. Store at room temperature (20–25 °C). Dilute 1:10 with ddH_2_O for 1 × TBS buffer.
- 1 × TBS + 0.05% Empigen: Add 83 ml of 30% Empigen into 500 ml of 1 × TBS buffer (Table 3).

- Second wash buffer solution:
  - 10 × phosphate-buffered saline (PBS): 20 PBS tablets and up to 950 mL of ddH_2_O. Adjust pH to 7.4 with HCl up to 1000 mL and purify with a 0.2 µm filter and store at room temperature (20–25 °C). Dilute 1:10 with ddH_2_O for 1 × PBS buffer.
- 10 × PBS buffer + 1% Tween. Add 10 ml of Tween 20 into 990 ml of 10 × PBS buffer. Store at room temperature (20–25 °C). Dilute 1:10 with ddH_2_O for 1 × PBS buffer + 0.1% Tween.

- Coating buffer and antibody:
  - 0.05 M NaHCO_3_: 4.2 g NaHCO_3_ and up to 450 mL of ddH_2_O. Adjust pH to 8.5 and add ddH_2_O up to a volume of 500 mL. Store at room temperature (20–25 °C).
- Anti-HIV Gag CA-p24: reconstitute according to manufacturer’s instructions. Make 100 µl aliquots and store at − 20 °C until use. To coat one 96-well plate, add 25 µl of aliquoted capture antibody into 2,475 µl of 0.05 M NaHCO_3_ (10 µg/mL) and dispense 25 µl of capture antibody solution into each well.

- CA-p24 standard protein and curve:
  - CA-p24 protein: reconstitute with 1 × TBS, 20% Sheep serum, and 1% Empigen to a concentration of 10 µg/mL. Dilute 1:10 with 1 × TBS + 1% Empigen to a concentration of 1 µg/mL. Make 30 µl aliquots and store at -80 °C until use.
  - CA-p24 standard curve: add 25 µl of aliquoted CA-p24 standard into 225 µl of dilution buffer (1 × TBS + 0.05% Empigen) (working stock). Prepare standard curve dilutions as specified in Additional File 2: Table S2.

- Detection antibody, conjugate, and substrate (**ABR**, **ANG**):
  - HIV-CA-p24, Alkaline Phosphatase Conjugate monoclonal antibody (AP-mAb): reconstitute and dilute 1:5 by adding 20 µl of antibody into 80 µl triethanolamine. Store at 4 °C until use.
  - Prepare the conjugate for one 96-well plate as followed: 2 mL of 1 × TBS + 0.5 mL of sheep serum + 2.5 µl of Tween 20 + 0.05 g of skim milk (2%) + 0.31 µl of the reconstituted conjugated (1:40,000, 60 ng/mL). Dispense 25 µl of conjugate solution into each well.

- Alkaline phosphatase substrate: dispense 25 µl of LumiPhos Plus solution (ready-to-use) into each well (Table 3).

2. Sino Biological (SB) and Anogen (ANG) systems

- Wash, blocking, and dilution buffers:
  - 10 × PBS buffer + 0.5% Tween: add 5 ml of Tween 20 into 990 ml of 10 × PBS buffer (as prepared in the **ABR** protocol). Store at room temperature (20–25 °C). Dilute 1:10 with ddH_2_O for 1 × PBS buffer + 0.05% Tween (Tables 4–5).
- 1 × PBS + 0.05% Tween + 2% bovine serum albumin (BSA): to prepare the blocking buffer needed for one 96-well plate, add 0.2 g of BSA into 10 ml of 1 × PBS buffer + 0.05% Tween.
- 1 × PBS + 0.05% Tween + 0.1% BSA: to prepare the dilution buffer needed for one 96-well plate, dilute 1:20 the blocking buffer into new 1 × PBS buffer + 0.05% Tween as needed.

- Coating antibodies:
  - Mouse Anti-HIV-CA-p24 (**SB**): reconstitute according to manufacturer’s instructions. To coat one 96-well plate, add 5.6 µl of coating antibody into 2.8 mL of 1 × PBS (2 µg/mL) and dispense 25 µl of coating antibody solution into each well (Table 4).
- Anti-HIV-CA-p24 mAb clone 340 (**ANG**): reconstitute according to manufacturer’s instructions. To coat one 96-well plate, add 25 µl of aliquoted coating antibody into 2,475 µl of 1 × PBS (10 µg/mL) and dispense 25 µl of coating antibody solution into each well (Table 5).

- CA-p24 standard curve:
  - CA-p24 standard curve: add 25 µl of aliquoted CA-p24 standard (ABR) into 225 µl of dilution buffer (1 × PBS + 0.05% Tween + 0.1% BSA) (working stock). Prepare standard curve dilutions as specified in Additional File 1: Table S3.

- Detection antibody, conjugate, and substrate:
  - Mouse Anti-HIV-CA-p24 horseradish peroxidase (HRP) antibody (**SB**): reconstitute according to manufacturer’s instructions. Dilute 1:1,000 in HRP stabilizer (Table 4) and store at 4 °C until use.
  - Conjugate (**SB**): to prepare the conjugate for one 96-well plate, dilute 1:1,000 aliquoted detection antibody by adding 3 µl of antibody into 3,000 µl of dilution buffer (100 ng/mL) and dispense 25 µl of conjugate solution into each well.
  - HRP substrate (**SB**): dilute 1:10 Lumiphos A and B solutions by adding 150 µl of solution A and 150 µl of solution B into 2.7 ml of ddH_2_O. Dispense 25 µl of LumiPhos A + B solution into each well (Table 4).

3. R&D system (RND)

- Wash buffer and blocking/dilution buffer:
  - 1 × PBS buffer + 0.05% Tween: dilute 1:10 10 × PBS buffer + 0.5% Tween (as prepared for **SB/ANG** protocols) with ddH_2_O.
  - 1 × PBS + 0.2% TritonX-100 + 1% BSA: to prepare the blocking/dilution buffer needed for one 96-well plate, add 0.4 g of BSA and 80 µl of TritonX-100 into 40 ml of 1 × PBS buffer (Table 6).

- Coating antibody:
  - Mouse Anti-HIV-1 Gag CA-p24 Capture Antibody: reconstitute according to manufacturer’s instructions with 0.5 mL of 1 × PBS buffer (Table 6). Make 45 µl aliquots and store at -20 °C until use. To coat one 96-well plate, add 41.8 µl of aliquoted coating antibody into 5 mL of 1 × PBS (4 µg/mL) and dispense 50 µl of coating antibody solution into each well.

- CA-p24 standard curve:
  - CA-p24 standard curve: add 5 µl of aliquoted CA-p24 standard (**ABR**) into 45 µl of dilution buffer (1 × PBS + 0.2% TritonX-100 + 1% BSA) (working stock). Prepare standard curve dilutions as specified in Additional File 1: Table S4.

- Detection antibody, conjugate, and substrate:
  - Biotinylated Mouse Anti-HIV-1 Gag CA-p24 Detection Antibody: reconstitute according to manufacturer’s instructions with 1.0 ml of dilution buffer (1 × PBS + 0.2% TritonX-100 + 1% BSA). Make 45 µl aliquots and store at − 20 °C until use. To prepare the antibody solution for one 96-well plate, add 41.8 µl of aliquoted detection antibody into 5 mL of 1 × PBS + 0.2% TritonX-100 + 1% BSA buffer (125 ng/mL) and dispense 50 µl of detection antibody solution into each well.
  - Conjugate: to conjugate the detection antibody in one 96-well plate, dilute 1:80 Streptavidin-HRP solution by adding 62.5 µl of antibody into 5 ml of 1 × PBS + 0.2% TritonX-100 + 1% BSA buffer and dispense 50 µl of solution into each well.
  - HRP substrate: dilute 1:10 Lumiphos A and B solutions by adding 300 µl of solution A and 300 µl of solution B into 5.4 ml of ddH_2_O (Table 6). Dispense 50 µl of LumiPhos A + B solution into each well.

**Table 3:** Key resources and reagents

Type	Reagent	Source	Identifier
Antibodies	Anti-HIV Gag CA-p24 (capture)	Aalto Bio Reagents	D7320
	HIV-CA-p24, Alkaline Phosphatase Conjugate monoclonal antibody (mAb) (detection)		BC1071-AP
	PBS tablets	Gibco	18912-014
	Tween 20	Merck	822184
Diluent solution, Blocking buffer	Empigen	Sigma-Aldrich	45165
	Skim Milk	SIAL	70166
Protein standard	HIV Gag CA-p24	Aalto Bio Reagents	AG 6054
Substrate solution	LumiPhos Plus	Lumigen	P-7000
96-well plates	Half-area white 96-well plates	Greiner	675074
Washer	BioTek ELx405 Select	Agilent	
Shaker	Microplate shaker	VWR	444-0270
Plate reader	GloMax® Navigator Microplate Luminometer	Promega	GM2010

**Table 4:** Key resources and reagents for the SB system

Type	Reagent	Source	Identifier
Antibodies	Mouse Anti-HIV-CA-p24 (capture)	Sino Biological	11695-MM08
	Mouse Anti-HIV-CA-p24 HRP conjugate (detection)		11695-MM15
Dilution buffer	PBS tablets	Gibco	18912-014
	Tween 20	Merck	822184
Protein standard	HIV Gag CA-p24	Aalto Bio Reagents	AG 6054
Conjugate	HRP Stabilizer	Abcam	ab270548
Substrate solution	Lumi-Phos HRP	Lumigen	PSA-100
96-well plates	Half-area white 96-well plates	Greiner	675074
Washer	BioTek ELx405 Select	Agilent	
Shaker	Microplate shaker	VWR	444-0270
Plate reader	GloMax® Navigator Microplate Luminometer	Promega	GM2010

**Table 5:** Key resources and reagents for the ANG system

Type	Reagent	Source	Identifier
Antibodies	HIV-CA-p24, Alkaline Phosphatase Conjugate monoclonal antibody (mAb) (detection)	Aalto Bio Reagents	BC1071-AP
	mAb anti-HIV-1 P24 (capture)	Anogen	MO-I40002D2
Dilution buffer	PBS tablets	Gibco	18912-014
	Tween 20	Merck	822184
Protein standard	HIV Gag CA-p24	Aalto Bio Reagents	AG 6054
Substrate solution	LumiPhos Plus	Lumigen	P-7000
96-well plates	Half-area white 96-well plates	Greiner	675074
Washer	BioTek ELx405 Select	Agilent	
Shaker	Microplate shaker	VWR	444-0270
Plate reader	GloMax® Navigator Microplate Luminometer	Promega	GM2010

**Table 6:** Key resources and reagents

Type	Reagent	Source	Identifier
Antibodies	Mouse Anti-HIV Gag CA-p24 Capture Antibody	R&D Systems	844,721, DY7360-05
	Biotinylated Mouse Anti-HIV Gag CA-p24 Detection Antibody	R&D Systems	844722, DY7360-05
Diluent solution, Blocking buffer	PBS tablets	Gibco	18912-014
	Tween 20	Merck	822184
	BSA	Roche	10735086001
	Triton X-100	Fisher	BP151-500
Protein standard	HIV Gag CA-p24	Aalto Bio Reagents	AG 6054
Conjugate	Streptavidin-HRP	R&D Systems	893975, DY7360-05
Substrate solution	Lumi-Phos HRP	Lumigen	PSA-100
96-well plates	Half-area white 96-well plates	Greiner	675074
Washer	BioTek ELx405 Select	Agilent	
Shaker	Microplate shaker	VWR	444-0270
Plate reader	GloMax® Navigator Microplate Luminometer	Promega	GM2010

### Statistical analyses

Microsoft Excel® 2016 was used to determine the standard curves for the ELISAs and to calculate the CA-p24 antigen concentrations in ng/mL. GraphPad v9.3.1 (GraphPad Software, Inc., USA) was used to analyze datasets and determine the sensitivity of the assays and Pearson’s *r* correlations between each assay. The statistical significance of differences were calculated using the Kruskal–Wallis’ test followed by a Benjamini–Hochberg test correction for multiple comparisons and two-way ANOVA followed by Tukey's post hoc test.

### Supplementary Information

**Additional file 1: Supplementary Figs. 1–4. (.pdf). Fig. S1.** Comparison of costs between in-house and commercial ELISA kits. **Fig. S2.** Standard curves generated by ABR-, ANG-, SB-, and RND-CA-p24 ELISAs. **Fig. S3.** Effect of viral inactivation on assay performance and within-assay reproducibility of ELISA systems. **Fig. S4.** Between-assay reproducibility of the ELISA systems.

**Additional file 2: Supplementary Tables 1–4 (.pdf). Table S1.** List of commercial ELISA kits and their costs. **Tables S2–S4.** Four tables with the volumes and details needed to prepare the standard curves for the ABR-, ANG-, SB-, and RND-CA-p24 ELISAs.

## Data Availability

All data generated or analyzed during this study are included in this published article and its supplementary information files.
