# Supplementary material for: In-house ELISA protocols for capsid p24 detection of diverse HIV isolates
Source: Virol J. 2023 Nov 17;20:269. doi: 10.1186/s12985-023-02242-5 (PMC10656996; doi:10.1186/s12985-023-02242-5)
Supplement: Supplementary file 1 — Additional file 1: Supplementary Figs. 1–4. (.pdf). Fig. S1. Comparison of costs between in-house and commercial ELISA kits. Fig. S2. Standard curves generated by ABR-, ANG-, SB-, and RND-CA-p24 ELISAs. Fig. S3. Effect of viral inactivation on assay performance and within-assay reproducibility of ELISA systems. Fig. S4. Between-assay reproducibility of the ELISA systems. [file 12985_2023_2242_MOESM1_ESM.pdf]

Additional File 1 for:

In-house ELISA protocols for capsid p24 detection of diverse HIV isolates

Mariano A. Molina[1], Monique Vink[1], Ben Berkhout[1], Elena Herrera-Carrillo[1,*]

[1]Department of Medical Microbiology, Laboratory of Experimental Virology, Amsterdam UMC, AMC, University of Amsterdam, Amsterdam, the Netherlands

*Correspondence: Elena Herrera-Carrillo, e.herreracarrillo@amsterdamumc.nl

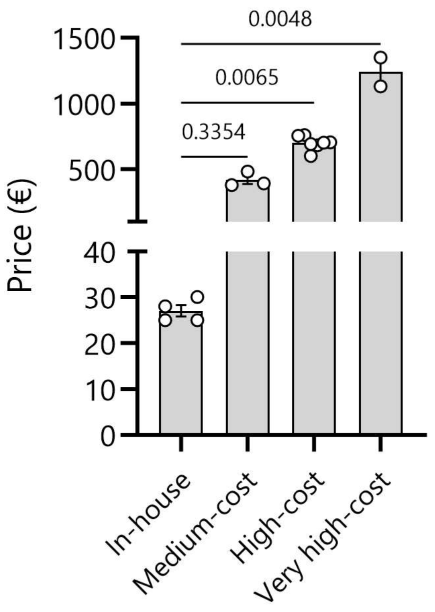

Figure S1. Comparison of costs between in-house and commercial ELISA kits. The costs per assay (one 96-well plate) for ABR-, ANG-, SB-, and RND-CA-p24 ELISAs are €25, €25, €30, and €28, respectively, including the costs for the reagents necessary for use (Tables 3-6). These costs are lower than medium-cost kits (mean €418) and significantly lower than high-cost (mean €699) and very high-cost kits (mean €1240). Differences in costs were analyzed by using a Kruskal-Wallis' test followed by the Benjamini-Hochberg test correction. *q* values are shown in the graph. *q* values < 0.10 are considered significant.

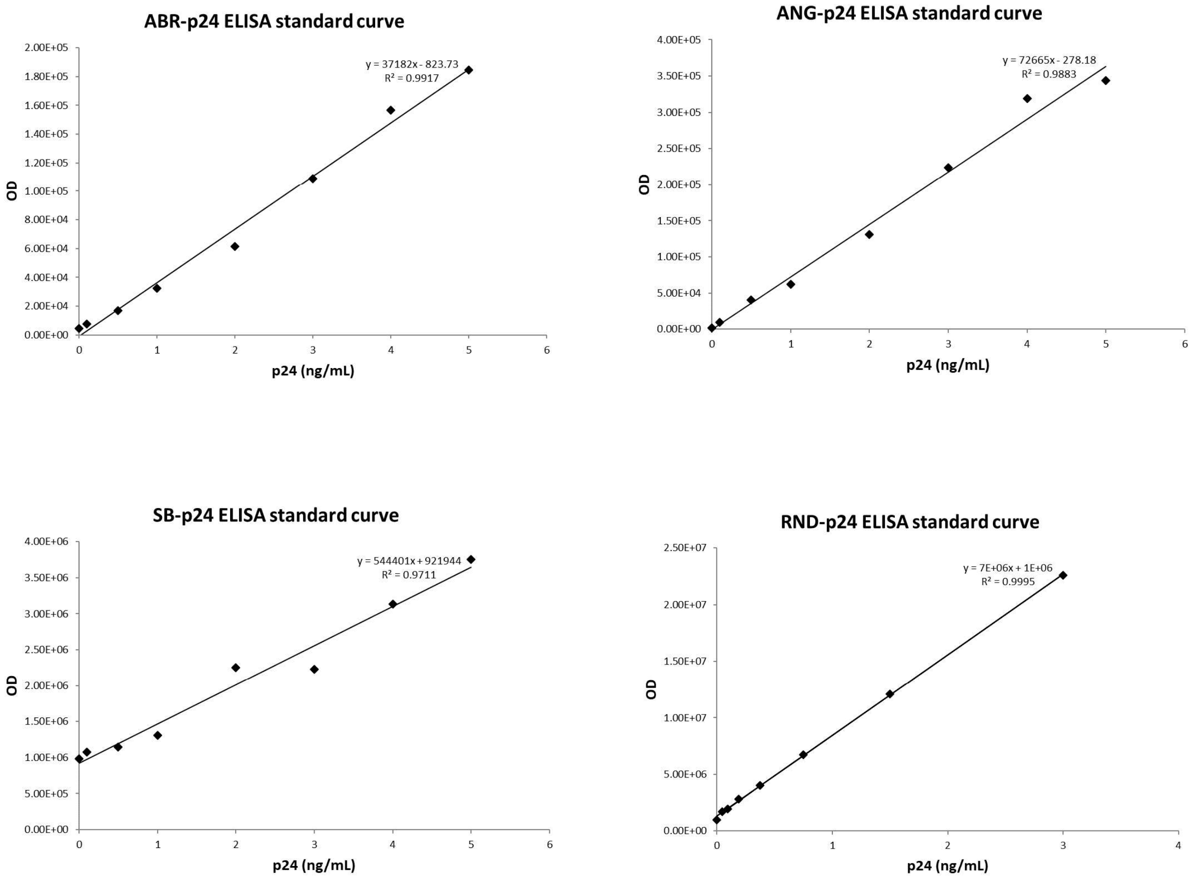

Figure S2. Standard curves for CA-p24 ELISAs. Standard curves generated by ABR-, ANG-, SB-, and RND-CA-p24 ELISAs demonstrate the detection limits of the assays. ABR-, ANG-, and SB-CA-p24 ELISAs can detect CA-p24 antigen up to 5 ng/mL, whereas RND-CA-p24 ELISA can detect CA-p24 antigen up to 3 ng/mL. The high correlation (R^2 ≥ 0.97) between CA-p24 protein standard concentrations and the measured optical density (OD) in all assays further show the accuracy of each system.

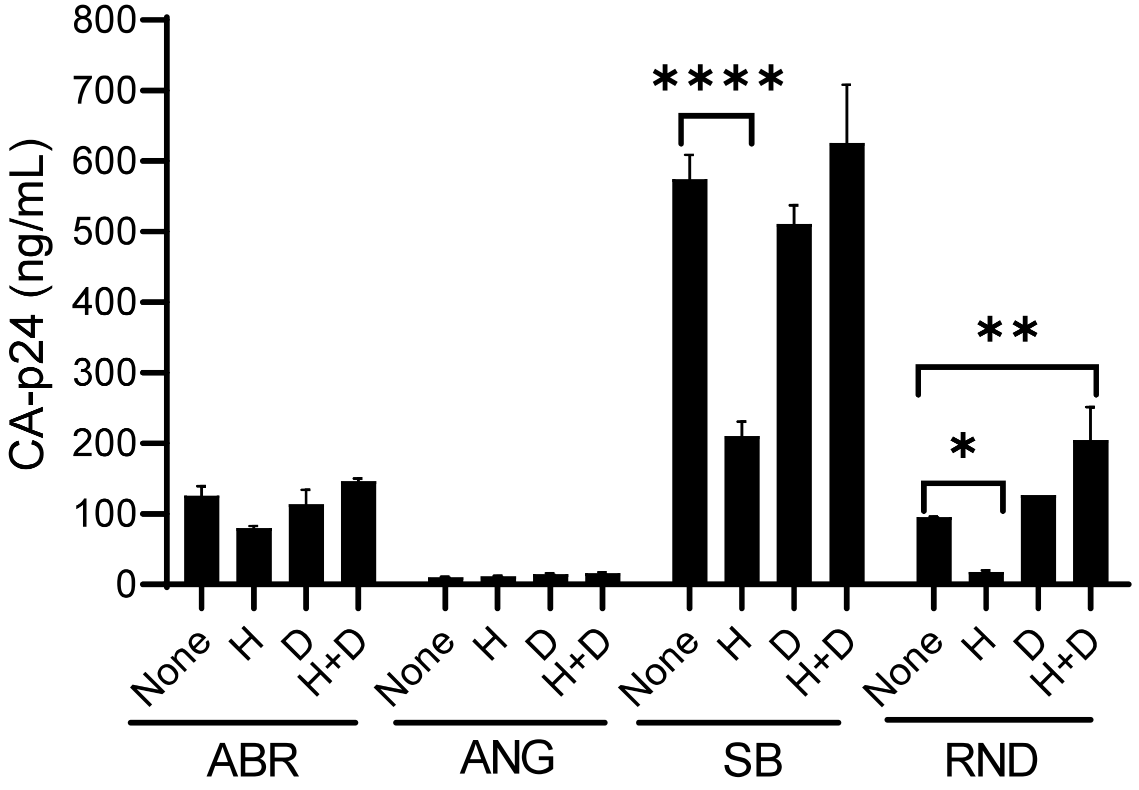

Figure S3. Effect of viral inactivation on assay performance and within-assay reproducibility of ELISA systems. The ABR-, ANG-, SB-, and RND-CA-p24 ELISAs were tested for the effect of heat (H) and detergent-inactivation (D) on their CA-p24 quantitation using the NL4-3 HIV-1 strain as a model. Four conditions were tested: heat- along detergent-inactivation (H+D), no heat- or detergent-inactivation (None), heat-inactivation alone (H), and detergent-inactivation alone (D). The data represent the mean ± standard deviation (SD) of two experiments. Statistical analyses (two-way ANOVA followed by Tukey's post hoc test were performed, and differences among groups were considered significant when the corresponding *p* value was less than 0.05 (* p <0.05; ** p <0.01; *** p <0.001; **** p <0.0001).

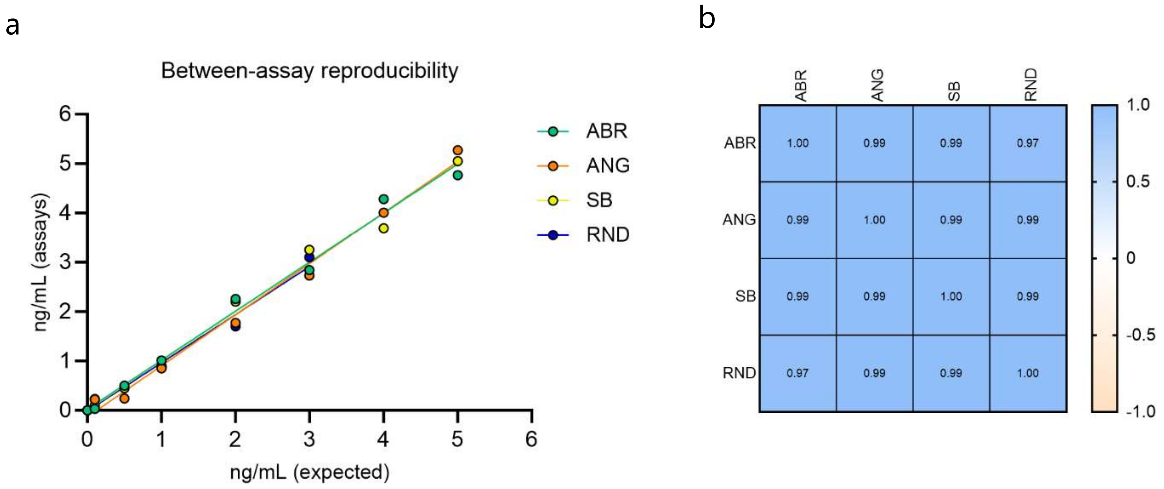

Figure S4. Between-assay reproducibility of the ELISA protocols. a. Analysis of the CA-p24 analysis and between-assay reproducibility by ABR-, ANG-, SB-, and RND-CA-p24 ELISAs using different concentrations of the CA-p24 standard protein (Tables 3-6). The detected CA-p24 concentrations by all assays corresponded to the expected concentrations in ng/mL. **b**. The CA-p24 measurements exhibited a significantly high positive correlation between all assays as tested by Pearson *r* correlation tests (p <0.002), demonstrating the between-assay reproducibility of the ELISA systems.
