# Supplementary material for: In-house ELISA protocols for capsid p24 detection of diverse HIV isolates
Source: Virol J. 2023 Nov 17;20:269. doi: 10.1186/s12985-023-02242-5 (PMC10656996; doi:10.1186/s12985-023-02242-5)
Supplement: Supplementary file 2 — Additional file 2: Supplementary Tables 1–4 (.pdf). Table S1. List of commercial ELISA kits and their costs. Tables S2–S4. Four tables with the volumes and details needed to prepare the standard curves for the ABR-, ANG-, SB-, and RND-CA-p24 ELISAs. [file 12985_2023_2242_MOESM2_ESM.pdf]

Additional File 2 for:

In-house ELISA protocols for capsid p24 detection of diverse HIV isolates

Mariano A. Molina[1], Monique Vink[1], Ben Berkhout[1], Elena Herrera-Carrillo[1,*]

[1]Department of Medical Microbiology, Laboratory of Experimental Virology, Amsterdam UMC, AMC, University of Amsterdam, Amsterdam, the Netherlands

*Correspondence: Elena Herrera-Carrillo, e.herreracarrillo@amsterdamumc.nl

Supplementary Table 1. List of commercial ELISA kits for comparison analyses.

Category	Source	Identifier	Price/96-well plate (€)
Medium cost	XpressBio	XBR-1000 Ext. Range	391.65
	XpressBio	XBR-1000	379.84
	Abnova	KA6284	482.52
High cost	R&D Systems	DHP240B	705.00
	RayBiotech	IQV-HIVP24-1	759.00
	abcam	ab218268	755.00
	TaKaRa	631476	681.00
	Origene	EA290002	600.00
	Biochain	Z7040001	691.84
	ZeptoMetrix	0801111	701.96
Very high cost	Arigo Biolaboratories	ARG82822_96wells	1131.00
	PerkinElmer	NEK050001KT	1348.72

Supplementary Table 2. Dilutions for the standard curve with the ABR system.

Dilutions	Dilution buffer	Working stock	CA-p24 concentration (ng/mL)
1	1 mL	0	0
2	900 µl	100 µl from 4	0.1 ng
3	900 µl	100 µl from 8	0.5 ng
4	990 µl	10 µl	1 ng
5	980 µl	20 µl	2 ng
6	970 µl	30 µl	3 ng
7	960 µl	40 µl	4 ng
8	950 µl	50 µl	5 ng

Supplementary Table 3. Dilutions for the standard curve with SB and ANG systems.

Dilutions	Dilution buffer	Working stock	CA-p24 concentration (ng/mL)
1	400 µl	0	0
2	800 µl	200 µl from 3	0.1 ng
3	500 µl	500 µl from 4	0.5 ng
4	500 µl	500 µl from 5	1 ng
5	980 µl	20 µl	2 ng
6	647 µl	20 µl	3 ng
7	480 µl	20 µl	4 ng
8	380 µl	20 µl	5 ng

Supplementary Table 4. Dilutions for the standard curve with RND system.

Dilutions	Dilution buffer	Working stock	CA-p24 concentration (ng/mL)
1	200 µl	0	0
2	200 µl	200 µl from 3	0.047 ng
3	200 µl	200 µl from 4	0.094 ng
4	200 µl	200 µl from 5	0.188 ng
5	200 µl	200 µl from 6	0.375 ng
6	200 µl	200 µl from 7	0.75 ng
7	200 µl	200 µl from 8	1.5 ng
8	388 µl	12 µl	3 ng
